# Prevalence of Common Comorbidities in Rheumatoid Arthritis in Rural New York Compared With National Data

**DOI:** 10.7759/cureus.19432

**Published:** 2021-11-10

**Authors:** Daniel T Fuller, Eyal Kedar, Carly R Lovelett, Sumona Mondal, Shantanu Sur

**Affiliations:** 1 Department of Mathematics, Clarkson University, Potsdam, USA; 2 Clinical and Rural Health Research, St. Lawrence Health, Potsdam, USA; 3 Department of Biology, Clarkson University, Potsdam, USA

**Keywords:** inflammation, hypertension, obesity, smoking, chronic obstructive pulmonary disease, arthritis

## Abstract

Background

Rheumatoid arthritis (RA) is a systemic autoimmune disease with multiple known comorbidities and risk factors. The rate and severity of different comorbidities among RA patients are influenced by various demographic, behavioral, and socioeconomic factors, which can vary widely between urban and rural areas. However, limited information is currently available regarding the association of comorbidities with RA in rural settings. In this study, we investigated the prevalence of common comorbidities and risk factors of RA among RA patients from a rural hospital located in rural northern New York and compared them against national patient records obtained from the National Hospital Ambulatory Medical Care Survey (NHAMCS).

Methodology

We compared de-identified patient records of 153 RA patients obtained from St. Lawrence Health (SLH) to 198 RA patients from the NHAMCS. After performing the descriptive analyses and removing outliers, two-sample tests of proportions were used for comparing the binary categories of sex, age, obesity, hypertension, chronic obstructive pulmonary disease (COPD), and congestive heart failure (CHF) between the two datasets. These analyses were applied to both weighted and unweighted sets of national data, and a p-value of <0.05 was considered statistically significant. The differences were then explored at a greater resolution by binning body mass index, blood pressure (BP), COPD prevalence, and tobacco usage data across different age groups.

Results

A significantly higher rate of diastolic hypertension (χ^2^ = 17.942, w = 0.232, p < 0.001) and over two times higher prevalence of COPD (χ^2^ = 7.635, w = 0.147, p = 0.006) were observed among RA patients in the rural group. The rates of CHF were significantly different only when sample weighting was applied. When categorized by age groups, diastolic BP showed a peak at 40-49 years, coinciding with the age group for high tobacco smoking and peak disease activity in rural RA patients.

Conclusions

A higher prevalence of comorbidities of RA such as hypertension (diastolic) and COPD are observed in patients from northern rural New York compared to the national average. Our findings indicate that rural RA patients might have a distinct comorbidity burden, suggesting the need for larger-scale studies.

## Introduction

Rheumatoid arthritis (RA) is a systemic autoimmune disease affecting approximately 0.5-1% of the population and is the most common disease observed in rheumatology clinics [1,2]. Although RA commonly presents as the characteristic inflammation of various target joints, it can affect multiple systems in the body. While several genes have been associated with the development of RA, both environment and lifestyle are important determinants of the incidence and severity of RA [3,4]. RA patients are at greater risk of developing several other diseases such as cardiovascular disease, interstitial lung disease (ILD) [5], chronic obstructive pulmonary disease (COPD), inflammatory eye disease, and depression [6-8]. The disease is more common and severe among the elderly and females although certain comorbidities such as ILD are more frequent among males [5,9]. Comorbidities have profound impacts on the disease activity of RA in the body, often substantially increasing pain and mortality. Several of these comorbid conditions are also known to increase the risk of developing RA and its progression [8,10,11]. The impact of lifestyle and behavioral factors on RA has been investigated, and tobacco smoking has been shown to have a strong association with RA, being responsible for approximately 35% of seropositive RA cases [12]. Additionally, socioeconomic status reportedly has a strong impact on RA rates, with a higher incidence of the disease found among populations with lower income and education [4].

While the risk factors and comorbidities of RA have been studied in detail, relatively little is known about the effects due to the factors specific to rural lifestyle and conditions. Research in the past two decades has provided a better understanding of rural health disparities, including the issues pertaining to access to medical care and population health [13]. Social, societal, and behavioral factors have a major influence on health and can differ substantially between rural and non-rural populations [13]. The rural population of the United States has a higher average age, lower physical activity, and increased rates of high-risk behaviors such as tobacco smoking [14]. In addition, rural environments are generally accompanied by lower education and increased poverty [15]. These factors are thought to contribute to a higher prevalence of many chronic diseases such as diabetes and coronary heart diseases among rural residents, as well as a widening gap in life expectancy between rural and urban populations [15,16]. Because many of these factors are known risk factors for RA, it raises the critical question of whether RA in rural settings has a poorer outcome [17]. This situation is further compounded by the severe shortage of the rheumatology workforce in rural areas and the long travel distance to access specialist care, causing a significant delay in diagnosis and treatment [18].

Despite the pressing need for attention, current literature on rural RA is extremely limited [17]. In this study, our goal is to better define the rural RA landscape in the United States and begin the process of identifying rural determinants of RA. In this study, we sought to compare a rural RA population to RA patients nationally using data from rural RA patients from St. Lawrence Health located in Saint Lawrence County (SLC). Although SLC is the largest county in New York by area, it is one of the poorest counties of the state, with nearly 20% of the population living below the federal poverty level. Markers of socioeconomic status for the county, such as education level and per-capita income, fall well below the national average. Furthermore, a recent report on county health rankings placed SLC at the 44th and 56th positions among a total of 62 counties in New York state in terms of health outcomes and health factors, respectively [19]. The rural location of SLC, its poor health ranking in the state, and a conspicuous disparity of socioeconomic status compared with the national average made it a suitable location to study RA patients. In this work, we focused on the prevalence of common comorbidities and risk factors among rural RA patients and compared the findings against the national dataset available from the National Hospital Ambulatory Medical Care Survey (NHAMCS). Additionally, the differences in the disease activity of RA across age groups were examined in the rural population.

## Materials and methods

Rural data

De-identified RA patient records were obtained from SLH. The records span four years (2016-2019) and include health measures and diagnoses from individual patient encounters at the rheumatology clinic in SLH. RA diagnosis was made following the American College of Rheumatology/European League Against Rheumatism Collaborative Initiative 2010 criteria. The raw data included information for single or multiple appointments for each patient, and therefore, a surrogate dataset was created by summarizing each measure of interest across all appointments using measures of central tendency. Means were used for continuous variables and modes were used for discrete variables. All continuous variables were filtered for extreme outliers that were more than three interquartile ranges (IQRs) above the 75th percentile or three IQRs below the 25th percentile. Participants below the age of 20 were excluded from this study.

National data

The NHAMCS is an annual cross-sectional survey that collects ambulatory care information from outpatient, emergency, and ambulatory surgery locations in non-institutional and short-stay hospitals nationwide. Because of the size of the target population, a stratified multistage random selection of ambulatory care visits is used to provide proper sampling (detailed information on the sampling design is available at Vital and Health Statistics; Series 2, No. 108 [20]). For each visitation, members of the staff complete a survey by listing detailed information about the patient, including demographics, the reason for visit, and current medication. Additionally, the survey includes attending physicians’ diagnoses, coded according to the International Classification of Disease, Ninth Revision, Clinical Modification (ICD-9-CM), and the services rendered by the hospital or clinic. We utilized combined data from 2008 to 2011 totaling 134,410 visits and filtered out all patients who did not have RA (ICD-9-CM: 714.0) listed in the “primary diagnosis,” “secondary diagnosis,” or “tertiary diagnosis” sections, or in the “reason for visit” section. Continuous variables for the national data were filtered for extreme outliers by IQR in an identical manner to the rural data.

Study measures

Demographic and Behavioral Data

The information on sex, age, and tobacco usage was obtained from patient charts for both local and national datasets. For measurements across age, the variable was categorized into groups of 10 years starting from 20 years. Tobacco usage was recorded as “yes” or “no” for the rural dataset and “current” or “not current” for the national dataset.

Physiological Variables

Information on systolic and diastolic blood pressure (BP) (mmHg), weight (kg), height (cm), and C-reactive protein (CRP, mg/dL) was obtained from patient charts for both local and national datasets. Additionally, component measures to quantify the Disease Activity Score with CRP (DAS28-CRP) were extracted from the rural data, which included tender joint 28-count (TJC), swollen joint 28-count (SJC), global health (GH) represented by self-assessed pain on the scale of 0-100, and CRP (mg/dL). DAS28-CRP was calculated following the established method using the following equation, and patients with a score of less than 2.6 were considered to have minimal disease activity (remission) [21].

$DAS28\! -\! CRP=0.56 \sqrt {TJC} \: +\: 0.28\sqrt {SJC}\: +\:0.014GH \: +\: 0.36ln(CRP+1)\: +\:0.96$

Comorbid Conditions

COPD status was recorded from patient charts for both local and national data. The SLH dataset also included patient data on ILD and congestive heart failure (CHF). Weight and height were used to calculate body mass index (BMI), which was then used to define obesity (BMI ≥ 30, obese; BMI < 30, not obese). Systolic and diastolic hypertension were defined by systolic BP and diastolic BP of ≥ 130 mmHg and ≥ 80 mmHg, respectively [22]. The history of anti-hypertensive drug use was not considered in the classification of hypertension.

Statistical analysis

Statistical analyses and visualizations were performed via R version 4.0.0. All plots were produced using the ggplot2 package. The NHAMCS survey design was implemented using the survey package in R which accounts for sample weights, sampling clusters, and the stratification component of the informative sampling, allowing for unbiased inference. We reported data and ran statistical tests both with raw counts and design-adjusted counts, which takes into account the sampling design due to the sampling strategy adopted in the NHAMCS. The chi-square (χ2) test for equality of proportions, which computes significant differences between the distributions of independent groups of nominal variables with mutually exclusive categories, was used to compare the characteristics of local and national RA patients. The χ^2^ test statistic, effect size (w), power (1-β), and p-values were reported. A p-value of <0.05 was considered significant.

Data availability

The national dataset used in the study is publicly available from the NHAMCS program website (https://www.cdc.gov/nchs/ahcd/index.htm). The rural RA dataset is available from the authors upon request.

## Results

Sample characteristics

**Figure 1:**
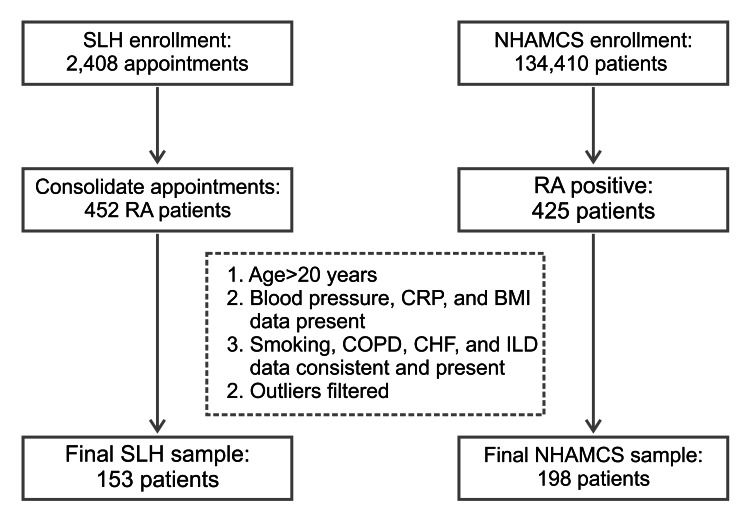
Flow diagram illustrating the selection of study population. RA: rheumatoid arthritis; SLH: St. Lawrence Health; NHAMCS: National Hospital Ambulatory Medical Care Survey; CRP: C-reactive protein; BMI: body mass index; COPD: chronic obstructive pulmonary disease; CHF: congestive heart failure; ILD: interstitial lung disease

The SLH sample consisted of 2,408 appointments for 452 RA patients. The NHAMCS national data had a sample size of 425 RA patients. Patients in both datasets were further filtered for age (>20 years included in this study), missing data, and outlying BP values, leaving 153 patients in the SLH (rural) group and 198 patients in the NHAMCS for comparative analysis (Figure 1).

Two-sample tests of proportion were conducted to compare the binary categories of sex, age, obesity, hypertension, tobacco usage, COPD, and CHF between rural and national datasets (Table 1). Rural RA patients had proportionally fewer females (χ^2^ = 8.990, w = 0.167, p = 0.003). Rural patients also had higher rates of both diastolic hypertension (χ^2^ = 17.942, w = 0.232, p < 0.001) and COPD (χ^2^ = 7.635, w = 0.147, p = 0.006). There were no significant differences between the two groups regarding age, obesity, systolic hypertension, tobacco usage, and CHF.

**Table 1:** Distribution of each category of grouping variable listed for the rural (SLH) and the national (NHAMCS) dataset. Chi-square (χ^2^) test statistic, effect size (w), power (1-β), and p-values are shown for statistical comparison; significant differences (p < 0.05) are shown in bold. Note that for certain variables, comparable data were not available in the NHAMCS dataset. SLH: St. Lawrence Health; NHAMCS: National Hospital Ambulatory Medical Care Survey; COPD: chronic obstructive pulmonary disease; CHF: congestive heart failure; ILD: interstitial lung disease; DAS28-CRP: Disease Activity Score with CRP; HTN: hypertension

Variable	Category	Rural (N = 153)	National (N = 198)	χ^2^	w	1-β	P-value
		N (%)	N (%)				
Sex	Female	104 (68.0)	163 (82.0)	8.990	0.167	0.878	0.003
	Male	49 (32.0)	35 (18.0)				
Age	50+	112 (73.2)	138 (70.0)	0.361	0.038	0.111	0.548
	20–49	41 (26.8)	60 (30.0)				
Obesity	Yes	71 (46.4)	109 (55.1)	2.248	0.086	0.362	0.134
	No	82 (53.6)	89 (44.9)				
Systolic HTN	Yes	72 (47.7)	100 (50.5)	0.284	0.034	0.098	0.594
	No	81 (52.3)	98 (49.5)				
Diastolic HTN	Yes	71 (46.4)	48 (24.2)	17.942	0.232	0.992	<0.001
	No	82 (53.6)	150 (75.8)				
Tobacco user	Current	39 (25.5)	41 (20.1)	0.867	0.057	0.185	0.352
	Not Current	114 (74.5)	157 (79.9)				
COPD	Yes	18 (11.8)	7 (3.5)	7.635	0.147	0.844	0.006
	No	135 (88.2)	191 (96.5)				
CHF	Yes	9 (5.9)	5 (2.5)	1.739	0.070	0.357	0.187
	No	144 (94.1)	193 (97.5)				
ILD	Yes	7 (4.6)	(2.2)	-	-	-	-
	No	146 (95.4)	(97.8)				
DAS28-CRP	2.6+	120 (78.4)	-	-	-	-	-
	0–2.599	23 (21.6)	-				

To assess the impact of the NHAMCS survey design on our analysis, we repeated the above statistical tests after adjusting for the sampling design (Table 2). Even after accounting for the sampling design, rural RA patients consistently showed a lower proportion of females (χ^2^ = 10.802, w = 0.004, p = 0.001), a higher proportion of diastolic hypertension (χ^2^ = 11.408, w = 0.004, p < 0.001), and COPD (χ^2^ = 15.063, w = 0.005, p < 0.001), while no difference was observed for systolic hypertension and tobacco usage. Interestingly, the rates of CHF were found to be significantly different between the two groups after the implementation of sample weighting (χ^2^ = 21.903, w = 0.006, p < 0.001), with the rural sample showing over a four-fold higher rate compared with the national sample. While correction for sampling weight is generally recommended for the analysis of NHAMCS complex survey data, the low effect sizes obtained using weighted data raise the issue of comparing weighted, large population-level data with small sample data. Taking into account this limitation and considering that overall results of the comparison between rural and national RA data after implementing the sampling design remained similar to the results without this correction (except for CHF, where a lesser precision occurred due to a low number of positives), we decided to conduct all further analyses involving the national data using its unweighted form.

**Table 2:** Percentage representation and statistical differences for each category of grouping variable listed for the SLH dataset and the design-adjusted NHAMCS dataset. Chi-square (χ^2^) test statistic, effect size (w), power (1-β), and p-values are shown for statistical comparison; significant differences (p < 0.05) are shown in bold. SLH: St. Lawrence Health; NHAMCS: National Hospital Ambulatory Medical Care Survey; COPD: chronic obstructive pulmonary disease; CHF: congestive heart failure; HTN: hypertension

Variable	Category	Rural (N = 153)	National (N ~ 690,000)	χ^2^	w	1-β	P-value
		N (%)	N (%)				
Sex	Female	104 (68.0)	549,278 (79.1)	10.802	0.004	0.923	0.001
	Male	49 (32.0)	145,085 (20.9)				
Age	50+	112 (73.2)	523,981 (75.5)	0.309	<0.001	0.100	0.579
	20–49	41 (26.8)	170,378 (24.5)				
Obesity	Yes	71 (46.4)	341,616 (49.2)	0.372	<0.001	0.106	0.542
	No	82 (53.6)	352,743 (50.8)				
Systolic HTN	Yes	72 (47.7)	329,833 (48.0)	<0.001	<0.001	0.051	0.977
	No	81 (52.3)	364,526 (52.0)				
Diastolic HTN	Yes	71 (46.4)	230,640 (33.2)	11.408	0.004	0.934	<0.001
	No	82 (53.6)	463,719 (76.8)				
Tobacco user	Current	39 (25.5)	165,297 (23.8)	0.155	<0.001	0.078	0.693
	Not Current	114 (74.5)	529,062 (76.2)				
COPD	Yes	18 (11.8)	33,032 (4.8)	15.063	0.005	0.983	<0.001
	No	135 (88.2)	661,327 (95.2)				
CHF	Yes	9 (5.9)	8,941 (1.3)	21.903	0.006	0.999	<0.001
	No	144 (94.1)	685,418 (98.7)				

Age distribution of major comorbidities

**Figure 2:**
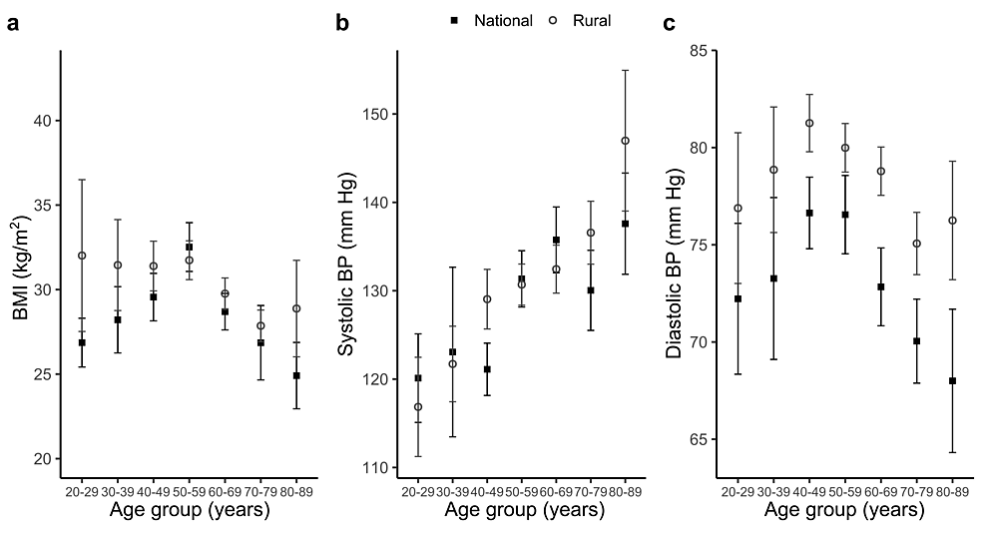
Comparison of (a) BMI, (b) systolic BP, and (c) diastolic BP among RA patients in rural and national datasets, distributed over different age groups. Data represent mean and standard deviation. BMI: body mass index; BP: blood pressure; RA: rheumatoid arthritis

To examine changes in RA comorbidities over time, we stratified them over age groups for both the rural and national data (Figure 2). Although the overall proportion of obesity did not differ significantly between the rural and RA sample population (Table 1), the distribution of BMI in different age groups revealed a distinct pattern. For the national data, BMI increased with age until it reached a peak at 50-59 years (average value: 33 kg/m^2^; Figure 2a), followed by a steady decrease with further advancement of age. However, rural RA patients exhibited higher BMI in the younger age groups, with average values remaining >30 kg/m^2^ across all age groups until 50-59 years, after which it followed a trend similar to the national sample only to show a late increase at 80-89 years. The trends of systolic BP were found to be similar in both groups, with average values ranging from 115 mmHg to 145 mmHg, and consistently increasing with age (Figure 2b). We observed that diastolic BP peaked at 40-49 years for both groups, but the average values at the peak for the national and rural RA patients were 77 mmHg and 81 mmHg, respectively (Figure 2c). Further, the difference in average diastolic BP varied across the age groups from 3 mmHg at 50-59 years to 9 mmHg at 80-89 years. This difference in diastolic BP across different age groups is consistent with a statistically significant difference in the prevalence of diastolic hypertension observed by the χ^2^ test for total sample populations (Table 1).

**Figure 3:**
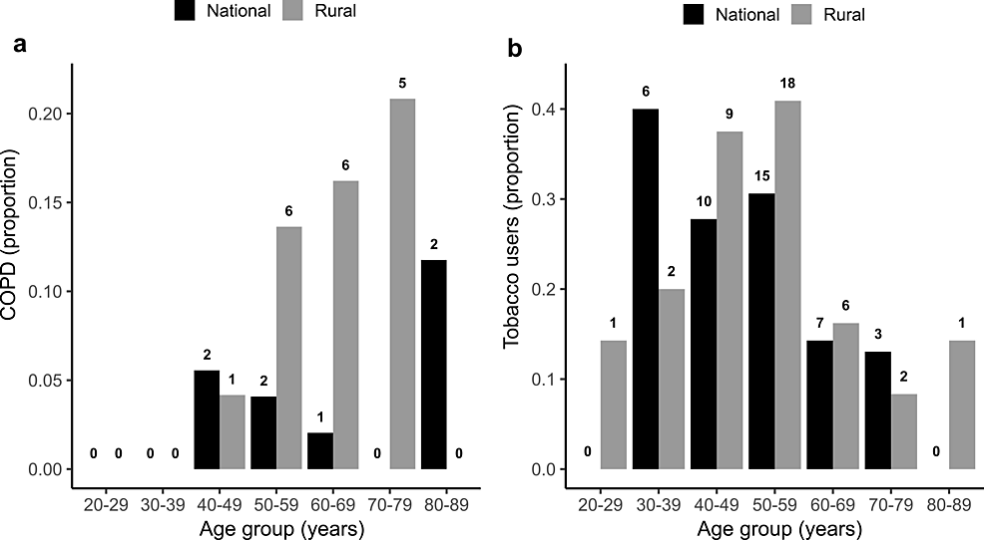
Comparison of (a) COPD and (b) tobacco usage between rural and national RA populations across age groups. The numbers above each bar represent the total number of individuals in each category. COPD: chronic obstructive pulmonary disease; RA: rheumatoid arthritis

COPD also demonstrated a distinct age-dependent change among rural RA patients (Figure 3). The prevalence of COPD increased steadily from 40-49 years, reaching a peak at 70-79 years, where 21% of rural RA patients were found to have COPD. Because tobacco smoking is a major risk factor of COPD, we compared tobacco usage between rural and national RA datasets across age groups. While a difference in tobacco usage was not found between overall populations (Table 1), we observed a higher usage among rural patients in the age groups from 40-49 to 60-69 years, with 40% of all rural RA patients in the age group of 50-59 years reporting tobacco use (Figure 3). Interestingly, these age groups also corresponded to the increased proportion of COPD observed among rural RA patients.

Rural disease activity across age groups

**Figure 4:**
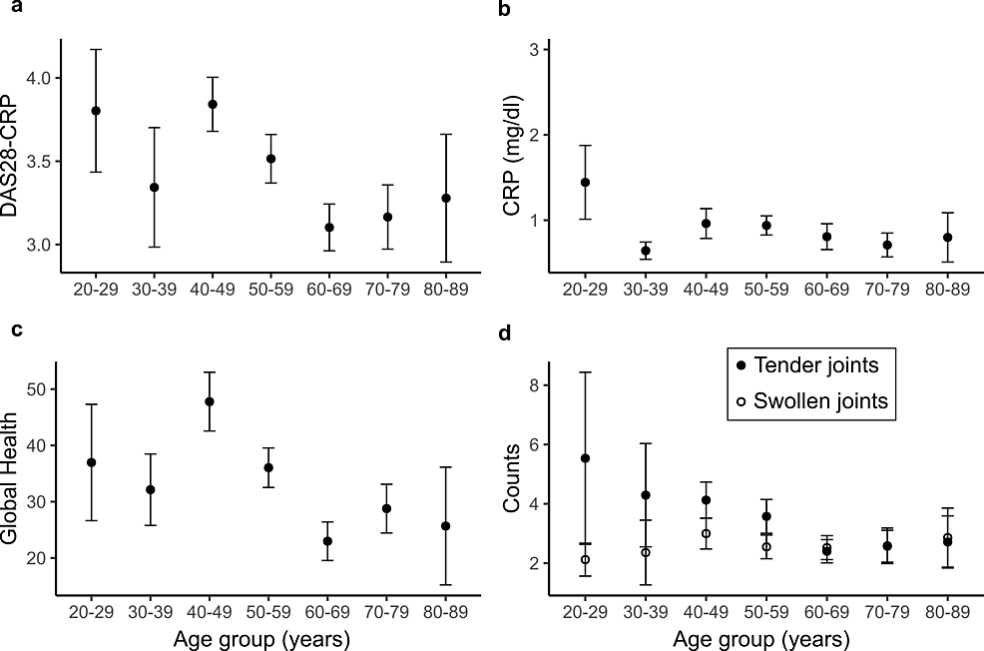
Distribution of DAS28-CRP and its components among different age groups in rural RA sample. Means and standard deviations of (a) DAS28-CRP and its components of (b) CRP level (mg/dL), (c) global health, and (d) swollen joint counts and tender joint counts plotted against age groups. CRP: C-reactive protein; DAS28-CRP: Disease Activity Score with CRP; RA: rheumatoid arthritis

To examine if RA disease activity in the rural sample varied among different age groups, we computed DAS28-CRP (Figure 4a). DAS28-CRP reached a peak at 40-49 years, with an average score of 3.8, followed by a fall with advancing age, except with a very late increase in the 80-89-year age group. Because DAS28-CRP is a composite disease activity measure, we further examined the age-dependent changes of individual components (Figures 4b-4d). Unlike DAS28-CRP, CRP level stayed almost the same across the age groups, with the maximum average value remaining within 1 mg/dL across all age groups (Figure 4b). In contrast, GH followed a pattern similar to that observed for DAS28-CRP, with the score increasing with age and reaching a peak value of 46 at 40-49 years, followed by a gradual fall (Figure 4c). The other two components of DAS28-CRP, SJC and TJC, showed a slightly different pattern across the age groups. The average TJC count decreased from approximately six to two over the age range of 20-29 years to 60-69 years, while the SJC count remained stable at two across all age groups (Figure 4d).

## Discussion

In this study, we compared a rural RA population in northern New York against a national population-based dataset of RA patients. Our results showed that the rural RA population under consideration has distinct demographic characteristics with a lower percentage of females compared with the national RA population. The patients in the rural RA population also displayed comparatively higher percentages of COPD and diastolic hypertension. While the differences in obesity and smoking rates, two potential risk factors of RA, were less prominent between the two patient populations, the age distributions for these conditions were markedly different. We also observed that disease activity in the rural RA population (assessed using the DAS28-CRP score) decreased across age after an initial peak at 40-49 years.

For both rural and national RA patients, systolic BP showed an increasing trend with age and diastolic BP reached a peak at the age of 40-49 years, followed by a gradual decrease, matching with the age-related changes reported for the general population [23]. Interestingly, the prevalence of diastolic hypertension is significantly higher in rural RA patients (46.4%) compared with the national counterpart (24.2%). Although the etiology for higher diastolic BP among rural RA patients is not evident from our data, limited access to care due to geographic and socioeconomic constraints impairing the control of hypertension could be a contributing factor. We speculate that the more prominent difference of diastolic BP between the rural and national RA population with advancing age could partially relate to a greater prevalence of COPD, which is known to increase the risk of hypertension [24]. Because RA is associated with a higher incidence of mortality and morbidity from cardiovascular diseases [25], the increased prevalence of diastolic hypertension could further increase the risk of cardiovascular complications in the rural RA population [26]. The coexistence of COPD and hypertension could further increase the cardiovascular risk of patients [27].

Among the RA comorbidities examined in this study, the difference in COPD prevalence was the most prominent between the rural (11.8%) and national (3.5%) datasets. Rates of tobacco smoking, a major high-risk behavior for COPD [28], were found to be higher among the age group of 40-49 years and above for the rural RA population, partly explaining this difference. Additionally, environmental factors might play a role in the increased COPD incidence observed in the rural dataset. The poorer rural communities in colder areas such as New York have a higher usage of indoor wood stoves and furnaces for heating which generate large amounts of wood smoke [29]. Multiple studies have shown that this smoke has detrimental effects on indoor occupants, including an increase in lung-related illnesses, especially COPD [30]. Chronic exposure to biomass-burning smoke has been shown to elicit an inflammatory response in the lungs similar to tobacco smoking [31]. Increased tobacco smoking and exposure to wood smoke suggest that both behavioral and environmental factors could contribute to higher COPD prevalence in SLC.

Additional rural-specific factors potentially contribute to the differences in the rates of RA comorbidities observed in our study. Demographic characteristics, behavioral practices, and socioeconomic disparities specific to rural SLC (Table 3) could play a role, alone or in combination. Both COPD and hypertension are positively associated with poor socioeconomic conditions such as low income and education [32,33]. Therefore, a lower per-capita income, a lower degree of education, and a higher percentage of non-elderly disability in SLC could increase the risk for COPD and hypertension. Beyond SLC, several of these factors are known to frequently exist in rural areas where an increased prevalence of COPD and a higher risk of mortality due to cardiovascular diseases have been reported [34,35].

**Table 3:** Comparison of demographic information between SLC and the entire nation. The values are based on data from the US Census Bureau from 2015 to 2019 (https://www.census.gov/quickfacts/fact/table/US/PST045219). SLC: Saint Lawrence County

	65+ years of age	White	Bachelor’s degree, aged 25+ years	Disabled, aged under 65 years	Per-capita income, past 12 months
United States	16.5%	76.3%	32.1%	8.6%	$34,103
SLC	18%	93.7%	23.8%	11.6%	$25,378

Disease activity measures are considered useful tools to facilitate treat-to-target strategy among RA patients in routine clinical care, and DAS28-CRP is one of the recommended measures [36]. We used DAS28-CRP to examine the RA disease activity among various age groups in the rural population. DAS28-CRP showed a peak at 40-49 years, followed by a gradual decrease at higher age groups, only to increase again at 80-89 years. GH, one of the DAS28-CRP components, also followed a similar trend with a peak at 40-49 years. Because the age group for the observed peak coincides with the common age for RA disease onset, we speculate this period represents more frequent acute flare-ups with higher perceived pain. The subsequent decrease or plateau phase likely corresponds to RA controlled under medication, while the increase in the disease activity observed at the very advanced age of 80-89 years is potentially due to overall lowered health combined with the progression of RA over a prolonged time. Higher inflammation during the initial disease manifestation and subsequent control under RA care could also explain the higher TJCs observed in younger patients and a gradual decrease in the count as the age advances.

The limitations of this study include a relatively small sample size and effect size, the lack of RA disease activity data in the urban patient population, and a potentially lesser diagnostic accuracy of RA in the national dataset. Additionally, due to constraints of data availability, the comparisons were made between patient populations from two different time periods (2016-2019 for rural data and 2008-2011 for national data), which could potentially contribute to bias. Despite these limitations, our study initiates the important process of better defining RA in the rural landscape, which would be critical for developing strategies for improving outcomes for rural RA patients.

## Conclusions

Our findings indicate that the prevalence of major comorbidities could be substantially different in rural regions compared to non-rural regions, and it is likely that a unique set of rural-specific factors, some of which potentially stem from rural-urban disparity, are the determinants of rural RA. Larger studies, ideally with data from multiple rural health systems, are needed to guide the diverse aspects of RA care in rural areas. Such an approach will require not only identifying the risk factors for RA disease activity and progression in rural areas but also strategies for training the rural RA workforce and for addressing the social determinants of both general and RA health in rural communities.
